# Leukocyte- and platelet-rich fibrin does not provide any additional benefit for tooth extraction in head and neck cancer patients post-radiotherapy: a randomized clinical trial

**DOI:** 10.4317/medoral.23804

**Published:** 2020-07-23

**Authors:** Luiz Felipe Palma, Marcelo Marcucci, Cíntia Maria Remondes, Leandro Chambrone

**Affiliations:** 1 PhD, MSc. Dentistry Program, Ibirapuera University. São Paulo, Brazil; 2 PhD. Discipline of Descriptive and Topographic Anatomy, Department of Morphology and Genetics, Federal University of São Paulo. São Paulo, Brazil; 3 PhD. Stomatology and Oral and Maxillofacial Surgery Center, Heliópolis Hospital. São Paulo, Brazil; 4 Stomatology and Oral and Maxillofacial Surgery Center, Heliópolis Hospital. São Paulo, Brazil; 5 Unit of Basic Oral Investigation (UIBO), School of Dentistry, Universidad El Bosque. Bogota, Colombia

## Abstract

**Background:**

One of the most important complications of radiotherapy (RT) for head and neck cancer (HNC) is osteoradionecrosis (ORN) of the jaws, arising mainly from tooth extractions. Thus, the present study aimed to evaluate the efficacy of leukocyte- and platelet-rich fibrin (L-PRF) in preventing ORN following tooth extraction in post-irradiated HNC patients, as well as other postoperative complications.

**Material and Methods:**

23 patients previously submitted to conventionally fractionated 3D-conformational RT for HNC underwent atraumatic tooth extractions with perioperative antibiotic therapy. Besides, they were randomly assigned to receive L-PRF clots to fill and cover the extraction sockets (n=11, Test Group) or not (n=12, Control Group). A visual analog scale was used to quantify postoperative pain on the 3rd and 7th days. For ORN diagnosis, patients were clinically assessed for up to 180 days. Other postoperative complications (edema, alveolitis, suture dehiscence, continuous bleeding, and oroantral communication) were also evaluated within this period.

**Results:**

No case of ORN or another surgical complication was observed and there were no differences in the postoperative pain scores between the groups on the 3rd and 7th days.

**Conclusions:**

L-PRF did not seem to provide any additional benefits than those achieved by the combination of the surgical and drug protocols used for tooth extractions in the post-irradiated HNC patients.

** Key words:**Radiotherapy, osteoradionecrosis, tooth extraction, head and neck cancer.

## Introduction

One of the most severe, debilitating, and well-known complications of radiotherapy (RT) is osteoradionecrosis (ORN) of the jaw (1), which was first reported about 100 years ago (2). Despite several definitions based mainly on clinical features, the most accepted one proposes the presence of exposed devitalized irradiated bone that fails to heal over a period of 3-6 months (3) and without local tumor recurrence (4).

ORN has been declining recently thanks to technological advances in Radiation Oncology (5) but prevalence rates as high as 22.9% have yet been reported (6). In this sense, tooth extraction is recognized as the main triggering factor for ORN; however, it may also occur spontaneously in the presence of residual foci of periodontal or periapical diseases or trauma resulting from poorly adapted prostheses (7,8).

Platelet concentrates, biological autologous products obtained from the patient's blood, have been widely applied in medical areas to enhance tissue healing and stimulate angiogenesis due to cytokines, growth factors, and other proteins released by platelets (9). Leukocyte- and platelet-rich fibrin (L-PRF), a second-generation platelet concentrate, has gained popularity in oral surgery also because of a slower, continuous release of growth factors when compared to other concentrates *in vitro* (10,11). Furthermore, the leukocytes presented in L-PRF may synthesize several pro- and anti-inflammatory cytokines as well (11).

On one hand, there are some promising case reports on the ORN treatment performed with platelet concentrates: 1) plasma rich in growth factors (9), 2) combination of platelet-rich plasma (PRP), alloplastic graft, and allogeneic dental pulp stem cells (12), 3) PRP gel (13), 4) platelet gel (14), and 5) combination of simvastatin and platelet-rich fibrin (15). On the other hand, a double-blind, split-mouth, randomized clinical trial showed that PRP was not effective in preventing ORN following tooth extraction pre-RT (16).

In light of these facts and considering mainly the noTable lack of evidence on this matter, the present study aims to evaluate whether the use of L-PRF could prevent ORN following tooth extraction in post-irradiated HNC patients, as well as the occurrence of other postoperative complications.

## Material and Methods

- Study design, ethical issues, and patient recruitment

This randomized clinical trial was conducted in the Stomatology and Oral and Maxillofacial Surgery Center at Heliópolis Hospital (São Paulo, Brazil) from August 2018 to November 2019 and adopting a convenience sample. The Research Ethics Committees of Ibirapuera University (#83264718.5.0000.5597) and Heliópolis Hospital (#82947318.4.0000.5449) had approved the study and then it was registered in the Brazilian Clinical Trials Registry – ReBEC (ID RBR-8y49vf [http://www.ensaiosclinicos.gov.br]).

Only patients requiring a single tooth extraction, 18 years of age or older, and presenting a history of conventionally fractionated 3D-conformational RT for HNC (cervicofacial and supraclavicular fossa fields) with total radiation dose between 60 and 70 Gy and no tumor local recurrence were considered eligible for inclusion in the study. The following exclusion criteria were also considered: systemic diseases or medications known to alter either healing processes (hard/soft tissues) or blood clotting, re-irradiation for recurrent tumors, and continuous use of medications for chronic pain.

- Study groups, clinical protocols, and procedures

Patients were randomly assigned to receive surgical and drug protocols either with L-PRF clots to fill and cover the extraction sockets (Test Group) or without it (Control Group). For that, randomization was performed using a computer-generated Table and the treatment code for each patient was allocated into a numbered, opaque, sealed envelope that was opened just before surgery.

The surgical protocol consisted of extraoral antisepsis with aqueous-based 2% chlorhexidine and intraoral with aqueous-based 0.2% chlorhexidine, vasoconstrictor-free local anesthesia with 3% Mepivacaine, and atraumatic surgical technique (careful syndesomotomy, no mucoperiosteal divulsion and osteotomy, tooth luxation and avulsion with forceps and elevators, vigorous curettage and copious socket irrigation with 0.9% saline solution, and the X suture technique with 4-0 monofilament nylon with no excessive tension). The drug recommendations were based on perioperative antibiotic therapy (Clindamycin 300mg every 8 hours for 10 days, with the first dose 3 days before the procedure) and postoperative pain medication, if necessary (Dipyrone - oral solution, 500mg every 6 hours, for up to 3 days).

- L-PRF obtaining and clot manipulation

**Figure 1:**
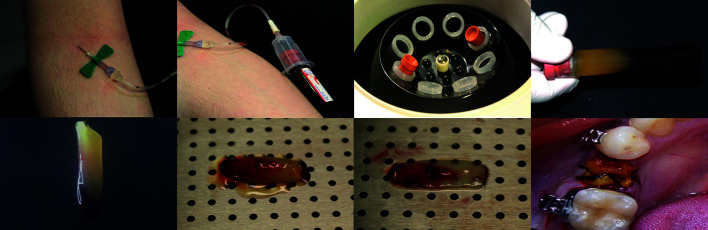
L-PRF clot obtaining, manipulation, and surgical application. From left to right and up to down: venipuncture; blood collection; centrifugation; L-PRF clot in a vacuum tube (red blood corpuscles at the bottom, platelet-poor plasma on the top, and fibrin clot in the middle); L-PRF clot isolated from the other blood components; L-PRF clot transferred to an especially designed kit; L-PRF clot after gentle compression; and a socket filled and covered with L-PRF clots.

The biomaterial was obtained moments before surgery by peripheral venipuncture (superficial veins of the upper limb). A 20 mL blood sample was collected from each patient through a 21G push-button blood set (Vacuette Safety Blood Collection Set + Luer Adapter™; Greiner Bio-one GmbH, Austria) with an appropriate holder (BD Vacutainer Single Use Holder™; BD, USA) into two vacuum tubes with clot activator (BD Vacutainer Serum Plus Blood Collection Tubes®; BD, USA). Immediately after the blood collection, both vacuum tubes were centrifuged (Daiki DT-4000 Centrifuge™; Ionlab Equipamentos Laboratoriais e Hospitalares Ltda, Brazil) under a ≅400 g relative centrifugal force for 12 minutes (17). L-PRF clots were then isolated and gently compressed using a sterile metal plate. Finally, the extraction socket was filled with one clot chopped and the other was used to cover the wound with stabilization by the X suture technique (Fig. 1).

- Evaluation of surgical outcomes

A visual analog scale composed of a horizontal line presenting values from 0 (no pain) to 10 (the worst pain imaginable) and cartoon faces with different expressions was given to the patients after surgery and instructions on it were also provided. They were asked about pain at the tooth extraction site on the 3rd day by phone call and in-person on the 7th day, during the appointment for stitch removal. Other postoperative complications such as edema, alveolitis, suture dehiscence, continuous bleeding, and oroantral communication were also evaluated. For ORN diagnosis, patients were clinically assessed for up to 180 days.

- Data synthesis

The patients’ demographic characteristics, individual habits, information on oncological treatments and tumors, and the tooth extraction outcomes were organized using the Excel™ software (Microsoft, USA) and then submitted to both descriptive and inferential statistical analyses by the Statistics Package for Social Sciences v21.0™ software (SPSS Inc., USA).

The Shapiro-Wilk test was used to check data distribution and the Student's T- and Chi-square tests were applied to identify differences between groups, with a P-value < 0.05 indicating statistical significance.

## Results

- Sample homogeneity

Twenty-three patients were involved in the study, totaling 12 in the Control Group and 11 in the Test Group.

The average age in the Control Group was 65.5 (±10.4) years (range 49-82) and in the Test Group was 59.2 (±8.1) years (range 49-79), with no statistically significant difference (Student's T-test; *P* = 0.122). Likewise, there was no difference (Chi-square test) between the groups regarding sex, skin color, tobacco use, alcohol use, primary tumor location, histological tumor type, total radiation dose, post-RT period, chemotherapy, and oncology surgery. Detailed data on the patients’ characteristics are in Table 1 and on the oncological treatments and tumors in Table 2.

**Table 1:**
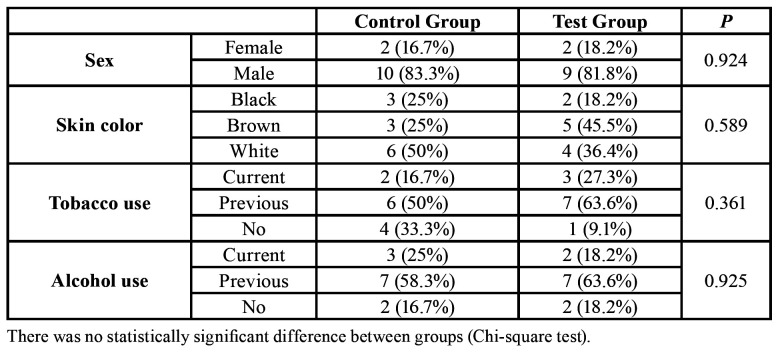
Patients’ additional data.

**Table 2:**
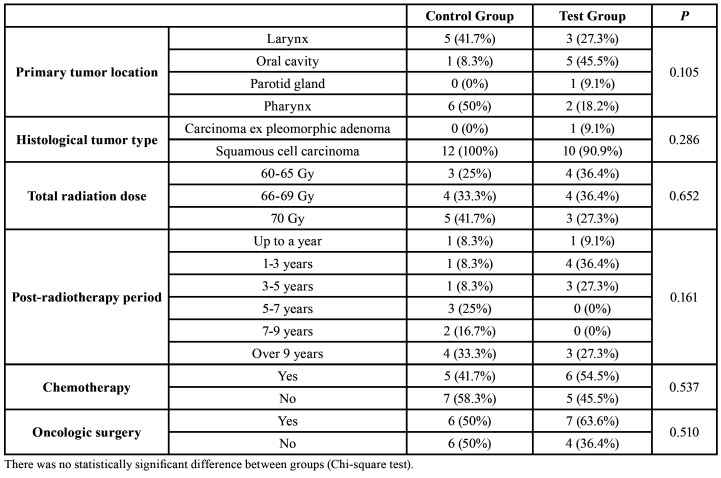
Patients’ tumors and oncologic treatments.

- Surgical procedures and outcomes

Eight upper (66.7%) and 4 lower teeth (33.3%) were extracted in the Control Group and 4 upper (36.4%) and 7 lower teeth (63.6%) in the Test Group, with no statistically significant difference (Chi-square test; *P* = 0.537). More information on the tooth extraction sites is presented in Table 3.

Only one patient from the Test Group experienced postoperative pain on the 3rd day, reporting a score of 2. Anyway, no difference between the groups was obtained as well (Student's T-test; *P* = 0.307).

No case of ORN development and postoperative complications was identified in any patient. The surgical outcomes are shown in detail in Table 4.

**Table 3:**
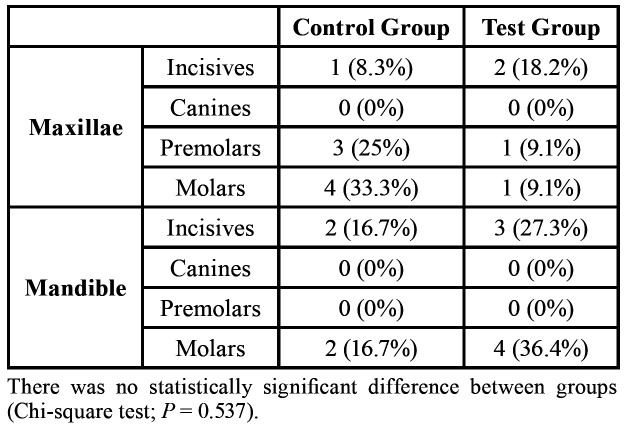
Tooth extraction sites.

**Table 4:**
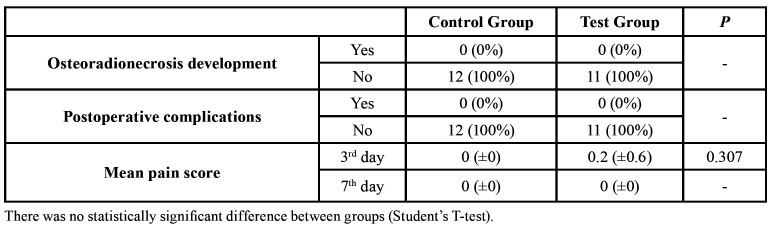
Surgical outcomes.

## Discussion

The present study aimed at investigating the use of L-PRF in tooth extraction sites of HNC patients post-RT, considering ORN development and other postoperative complications. The results from both study groups were highly satisfactory and may be considered of great clinical relevance due to the difficulties in the treatment of ORN and the related symptoms (e.g., localized mild algesia, dysesthesia, halitosis, severe pain, orocutaneous fistulas, and even pathological fractures) (18).

The design and hypothesis of the current study were mainly based on the likely benefits of L-PRF for ordinary tooth extractions in healthy patients: soft tissue and bone healing improvement, inflammation control, and postoperative pain reduction (19). These effects are thought to occur as L-PRF is a rich source of growth factors, cytokines, and other proteins (e.g., transforming growth factor β, platelet-derived growth factor, and vascular endothelial growth factor), which may impact positively on healing processes such as angiogenesis and immune control (20). Moreover, L-PRF seems to induce and stimulate the proliferation of osteoblasts, fibroblasts, and keratinocytes, as well as to promote an important differentiation of osteoblasts (19). Further advantages include mechanical protection to surgical wounds, ease of preparation and manipulation, and low cost (20).

Considering the probable role of microorganisms in ORN pathophysiology, perioperative systemic antibiotic administration might be rationale and was adopted here as a protocol; however, several different drug regimens and success rates have already been reported in the literature (8,21). Although there has been not enough evidence to support the prevention of alveolitis and infections in non-irradiated patients and taking into account all possible adverse effects, any minor clinical benefit would justify the use of prophylactic antibiotic therapy because of ORN morbidity and severity (22). Moreover, antibiotics are easy to administer and widely available (7).

Despite the conflicting results of other studies and a slight preference for a drug from the penicillin family due to the affectivity against most oral bacteria (22), clindamycin was administered. It is active against most strains of *Staphylococcus aureus*, several other gram-positive cocci, and gram-negative anaerobic pathogens (23), and is a good option for β-lactam-allergic patients. Clindamycin also provides good penetration into bone tissue, has been historically successful in the treatment of osteomyelitis (23), and is available for free in the Brazilian Public Health System.

Atraumatic surgical technique, alveoloplasty to remove bone spicules, primary wound closure without tension, a reduced number of teeth extracted per session (24), minimal periosteum divulsion, vasoconstrictor-free local anesthesia, and mouthwashes with chlorhexidine have been proposed as preventive factors for ORN following tooth extractions (8). Most of them were applied in this study, except for alveoloplasty and primary wound closure (avoiding periosteal divulsion or excessive tension at the edges) and mouthwashes with antiseptic solutions (aiding to maintain clot stability into the extraction sockets and the L-PRF clots over the surgical wounds).

To the best of the authors’ knowledge, there is no similar study on L-PRF in combination or not with surgical and drug protocols for tooth extractions in HNC patients post-RT. Although the current results are encouraging, they should be interpreted with caution mainly because of the limited sample size. Likewise, some factors that would increase the risk of ORN such as the period between RT completion and tooth extraction (25), concomitant chemotherapy (26), current alcohol and tobacco use (4,5), and the lack of accurate data on the total radiation dose delivered to each tooth extraction site (21) can be considered study bias.

The authors, however, in an attempt to mitigate the discrepancy among demographic characteristics of the study individuals, recruited only those who underwent conventionally fractionated 3D-conformal RT with cervicofacial and supraclavicular fossa fields, giving strong evidence that the extraction sites received substantial radiation total doses. As such, patients submitted to other techniques (e.g., hyperfractioned regimen, brachytherapy, intensity-modulated RT) were not considered for the analyses. Furthermore, the lack of any statistically significant difference between both groups regarding the patients’ variables indicates an adequate sample homogeneity.

In summary, within the limitations of this study, the use of L-PRF in tooth extractions for HNC patients post-RT did not seem to offer any additional benefit over to the surgical and drug protocols proposed.
